# Supplementary material for: A Soluble Platelet-Derived Growth Factor Receptor-β Originates via Pre-mRNA Splicing in the Healthy Brain and Is Upregulated during Hypoxia and Aging
Source: Biomolecules. 2023 Apr 21;13(4):711. doi: 10.3390/biom13040711 (PMC10136073; doi:10.3390/biom13040711)
Supplement: Supplementary file 1 [file biomolecules-13-00711-s001.zip › PAYNE-Biomolecules_rev2--RAW_BLOTS.pdf]

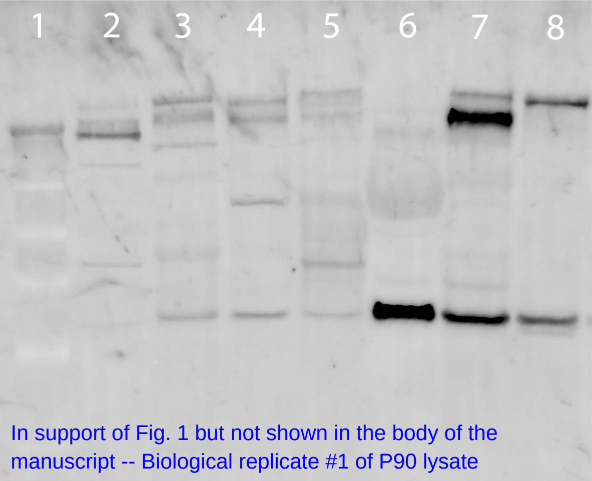


PDGFRb

1 - Marker
2 - Skeletal Muscle
3 - Heart
4 - Intestine
5 - Liver
6 - Serum
7 - Kidney
8 - Brain

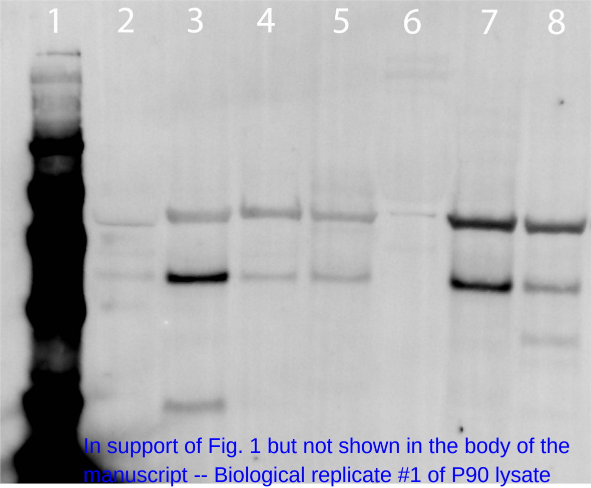


**Beta actin**

1 - Marker
2 - Skeletal Muscle
3 - Heart
4 - Intestine
5 - Liver
6 - Serum
7 - Kidney
8 - Brain

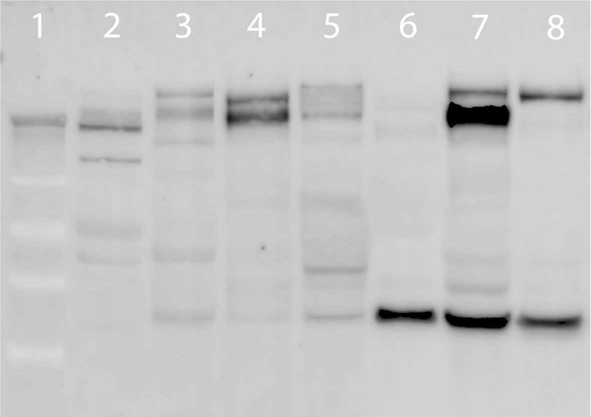


In support of Fig. 1 and shown in the body of the manuscript
-- Biological replicate #2 of P90 lysate

**PDGFRb**

1 - Marker
2 - Skeletal Muscle
3 - Heart
4 - Intestine
5 - Liver
6 - Serum
7 - Kidney
8 - Brain

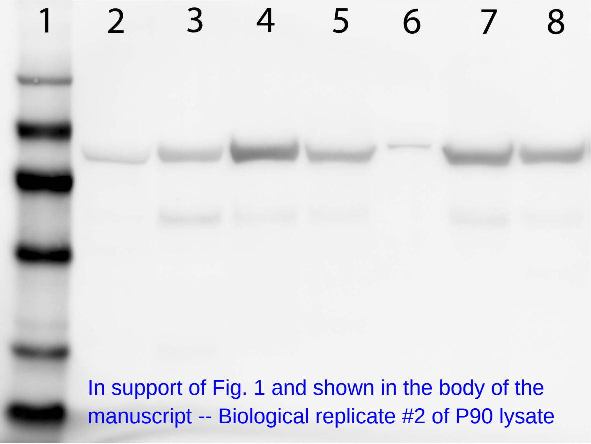


**Beta actin**

1 - Marker
2 - Skeletal Muscle
3 - Heart
4 - Intestine
5 - Liver
6 - Serum
7 - Kidney
8 - Brain

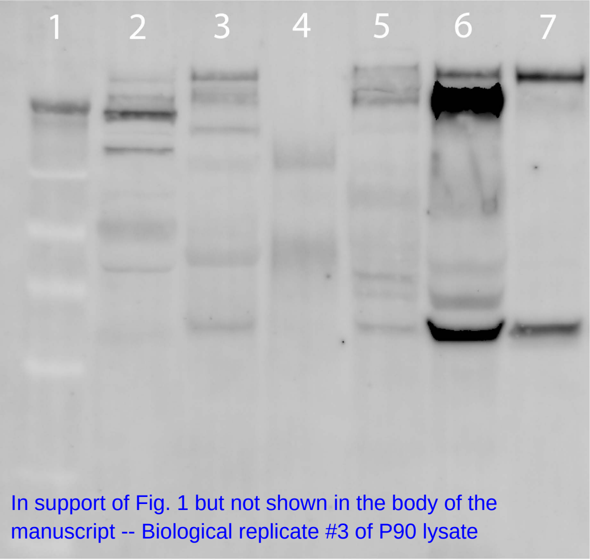


**PDGFRb**

1 - Marker
2 - Skeletal Muscle
3 - Heart
4 - Intestine
5 - Liver
6 - Kidney
7 - Brain

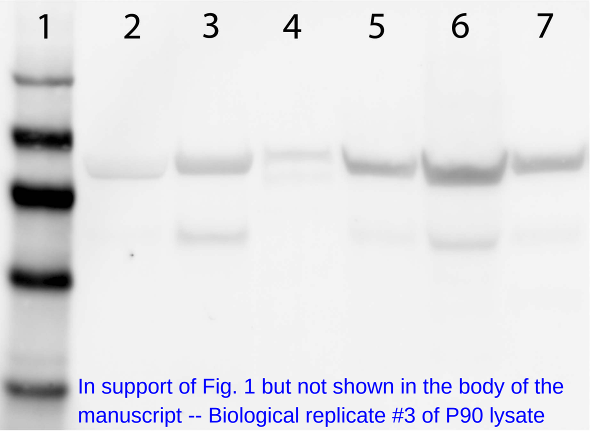


**Beta actin**

1 - Marker
2 - Skeletal Muscle
3 - Heart
4 - Intestine
5 - Liver
6 - Kidney
7 - Brain

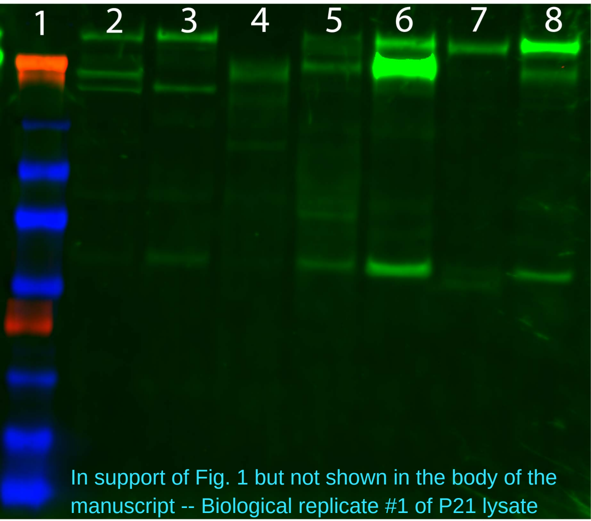


**PDGFRb**

1 - Marker
2 - Skeletal Muscle
3 - Heart
4 - Intestine
5 - Liver
6 - Kidney
7 - Brain
8 - Lung

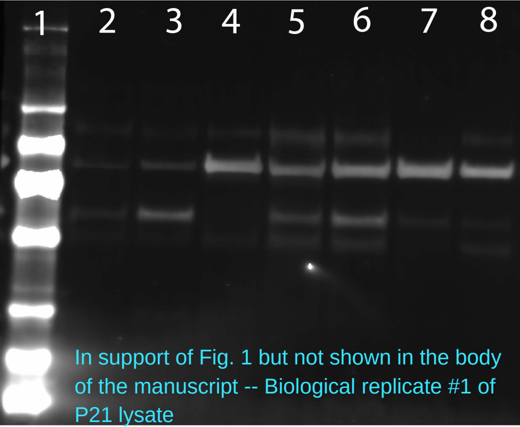


**Beta actin**
(45 kDa)

1 - Marker
2 - Skeletal Muscle
3 - Heart
4 - Intestine
5 - Liver
6 - Kidney
7 - Brain
8 - Lung

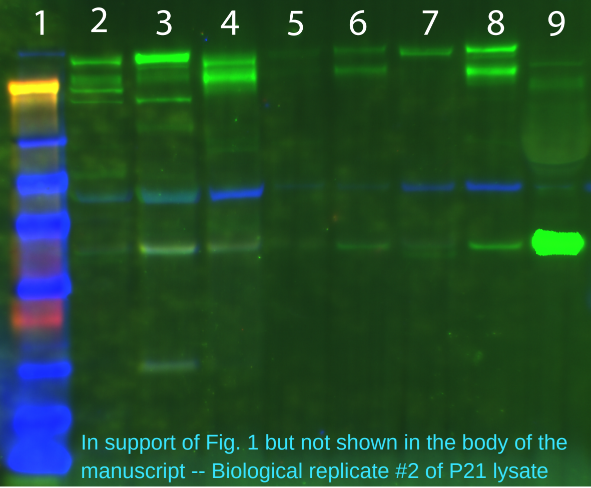


**PDGFRb**

**Beta actin**

1 - Marker
2 - Skeletal Muscle
3 - Heart
4 - Intestine
5 - Liver
6 - Kidney
7 - Brain
8 - Lung
9 - Serum

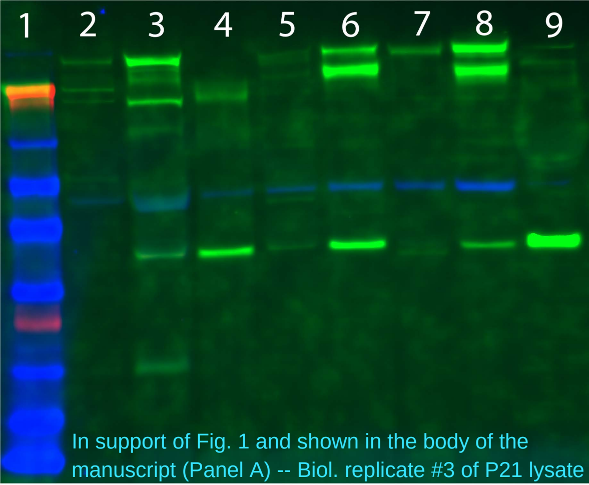


**PDGFRb**

**Beta actin**

1 - Marker
2 - Skeletal Muscle
3 - Heart
4 - Intestine
5 - Liver
6 - Kidney
7 - Brain
8 - Lung
9 - Serum

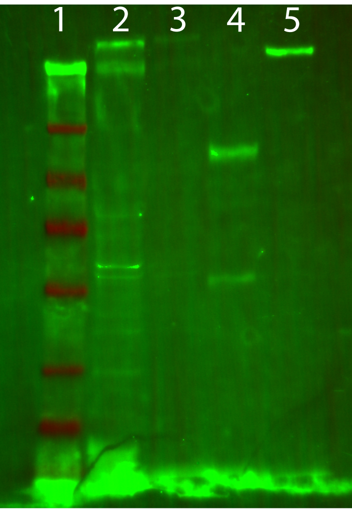


1 - Marker
2 - Whole Brain Lysate
3 - Depleted Lysate
4 - Co-IP Sample
5 - Recombinant PDGFRb

**PDGFRb**

in support of Fig. 3

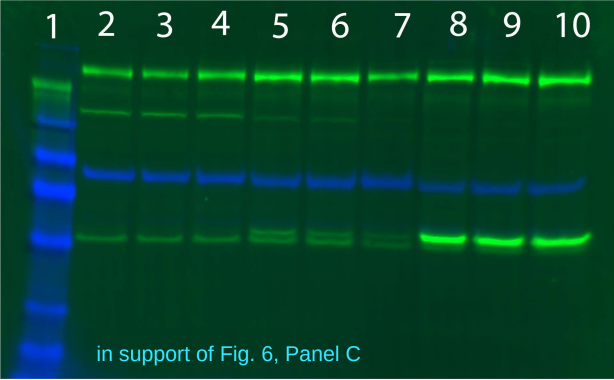


PDGFRb
Beta-actin

1 - Marker
2 - P7 Replicate 1
3 - P7 Replicate 2
4 - P7 Replicate 3
5 - P21 Replicate 1
6 - P21 Replicate 2
7 - P21 Replicate 3
8 - P90 Replicate 1
9 - P90 Replicate 2
10 - P90 Replicate 3

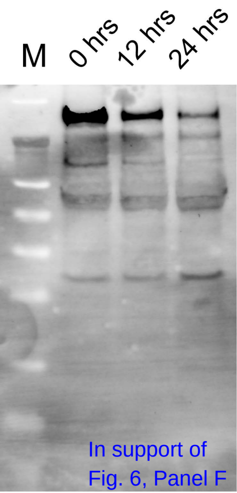


PDGFRb

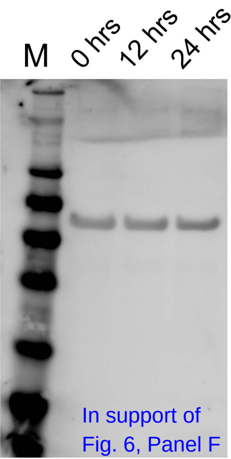


ßactin - stripped after PDGRB detection and reprobed
