# Supplementary material for: A Soluble Platelet-Derived Growth Factor Receptor-β Originates via Pre-mRNA Splicing in the Healthy Brain and Is Upregulated during Hypoxia and Aging
Source: Biomolecules. 2023 Apr 21;13(4):711. doi: 10.3390/biom13040711 (PMC10136073; doi:10.3390/biom13040711)
Supplement: Supplementary file 1 [file biomolecules-13-00711-s001.zip › PAYNE-Biomolecules_rev2--Supp_Info_Fig-v01.pdf]

Supplementary Materials and Methods

Immunohistochemistry and Confocal Microscopy

Normal WT brains (C57BL/6 – P21, n=3, both sexes) collected for immunohistochemistry and confocal microscopy were collected in a similar manner as described in the main article text for protein collection (see Materials and Methods) with the exception of intracardiac perfusion of 4% paraformaldehyde (in PBS) following PBS perfusion. Prior to sectioning, the olfactory bulbs and cerebellum were removed from the cerebrum using a single-edged razor blade (Garvey, 40475). Brains were then affixed caudally by superglue (Loctite, 1364076) to the vibratome dish with their ventral surface supported by a block of 4% agarose. They were then submerged in PBS and sectioned into 100 µm slices using half a double-edged razor blade (Electron Microscopy Sciences, 72000) in a vibratome (1000-Plus, Pelco 102, Ted Pella Inc.). Slices were transferred to a 24-well plate and then stored in PBS at 4°C until processing for immunohistochemistry.

Brain sections were blocked for 1 hour at room temperature in PBS and 0.1% Triton 100x (PBS-T) with 1.5% Normal Donkey Serum (Jackson ImmunoResearch, 017-000-121). Slices were then incubated at 4°C overnight in 1.5% Normal Donkey Serum in PBS-T with rat anti-PDGFR-β antibody (Affymetrix, 14-1402, 1:500) and goat anti-CD31/platelet-endothelial cell adhesion molecule-1 (PECAM-1) antibody (R&D Systems, AF3628,1:500) on a shaker. After incubation with primary antibodies, the slices were washed four times for 15 minutes at room temperature in PBS-T on a shaker.
Brain slices were then incubated either 4 hours at room temperature or overnight at 4°C in 1.5% Normal Donkey Serum in PBS-T with donkey anti-rat DyLight 550 (ThermoFisher, SA5-10029, 1:1000), donkey anti-goat AlexaFluor488 (Jackson ImmunoResearch, 705-545-147, 1:500), and 4',6-Diamidino-2-phenylindole (DAPI, Sigma, D9542, 1:1000). After incubation with secondary antibodies, the slices were washed four times for 15 minutes at room temperature in PBS-T, and then washed again 15 minutes at room temperature in PBS after final incubations of staining combinations. Stained brain slices were mounted in 50% glycerol in PBS, a coverslip applied (22 mm x 22 mm – 1.5 thickness, ThermoFisher, 12-541-B), and sealed with clear nail polish (Electron Microscopy Sciences, 72180). Images of stained brain sections were acquired with a Zeiss LSM 880 confocal microscope using a 20x objective and Zen Black software.

Subcortical regions were identified as being above ventricles and included cortex and subcortical white matter. Images were collected in 2x2 tile scans with 10% overlap in 50-100 z-axis sections as determined by optimal z-axis distance. Using Zen Black image processing capability, the original tile scans were stitched

together. Selected brain regions of interest were outlined and saved as new image files for analysis.

Supplemental Figures

Supplemental Figure S1. Immunofluorescent Labeling of PDGFRβ in the Mouse Brain Revealed Signals Alongside PECAM-1-Positive Vessels but also Distributed Diffusely throughout the Parenchyma. (A) Representative confocal images of a P21 male mouse brain sections (biological replicate #2) immunohistochemically labeled for PECAM-1 (i; green in iv) and PDGFRβ (ii; red in iv) with cell nuclei stained with DAPI (iii; blue in iv). Scale bars are 100 microns. Note the diffuse signal associated with PDGFRβ immunostaining in addition to signals associated with vessels in ii and iv. (B) Representative confocal images of a P21 female mouse brain sections (biological replicate #3) immunohistochemically labeled for PECAM-1 (i; green in iv) and PDGFRβ (ii; red in iv) with cell nuclei stained with DAPI (iii; blue in iv). Scale bars are 100 microns. Note the diffuse signal associated with PDGFRβ immunostaining in addition to signals associated with vessels in ii and iv.

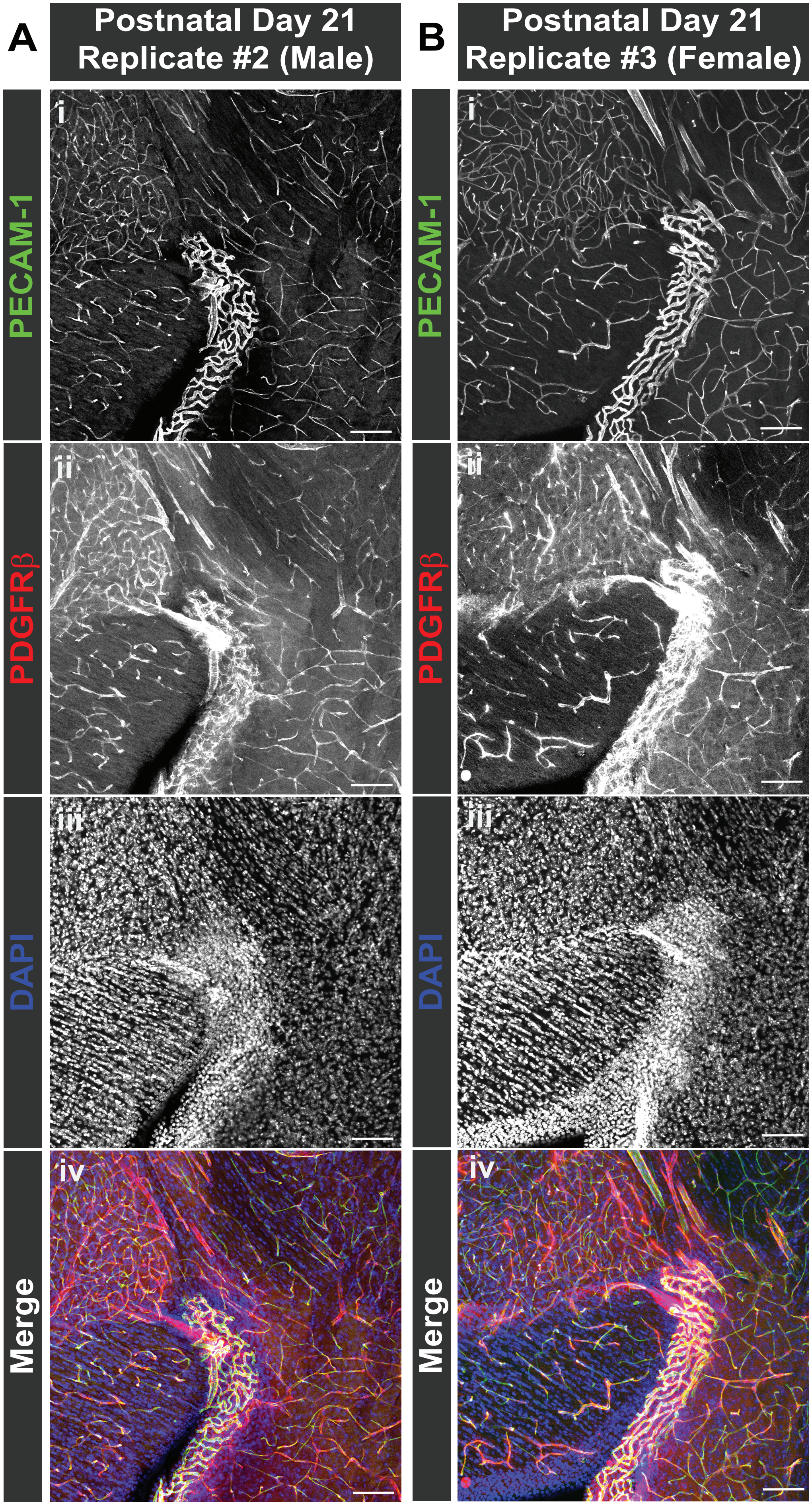

Supplemental Figure S1
